# Feasibility, acceptability, and outcomes of a pilot intervention facilitating communication about family building between patients with inherited cancer risk and their partners

**DOI:** 10.1016/j.pecinn.2022.100055

**Published:** 2022-06-01

**Authors:** Marleah Dean, Jonathan T. Baker, Maija Reblin, Elizabeth A. Hintz, Susan T. Vadaparampil, Carolyn Haskins, Gwendolyn P. Quinn

**Affiliations:** a Department of Communication, University of South Florida, Tampa, FL, USA; b Department of Health Outcomes and Behavior, Moffitt Cancer Center, Tampa, FL, USA; c Department of Family Medicine, University of Vermont, VT, USA; d Department of Communication, University of Connecticut, Storrs, CT, USA; e Department of Genetic Counseling, Moffitt Cancer Center, Tampa, FL, USA; f Department of OB-GYN, Grossman School of Medicine, New York University, NY, USA

**Keywords:** Inherited cancer risk, Family building, Couples, Discussion task, Interpersonal communication, Pilot intervention

## Abstract

**Objective:**

This study reports the feasibility, acceptability, and outcomes of a longitudinal, communication pilot intervention for patients with inherited cancer risk and their partners.

**Methods:**

Couples were recruited through social media and snowball sampling. At Time 1 and 2, 15 couples completed a structured discussion task about family building concerns and decisions, followed by an online post-discussion questionnaire and dyadic interview to provide feedback about the experience. Interview data were analyzed to assess outcomes using applied thematic analysis.

**Results:**

Participants reported the intervention created an opportunity for honest disclosure of family building goals and concerns. Participants also stated the structured nature of the discussion task was useful and did not cause additional stress. The intervention ultimately aided at-risk patients and their partners to realize their concordant concerns, discover/confront discordant concerns, and mutually agree upon next steps.

**Conclusions:**

This pilot intervention is feasible and acceptable. Furthermore, it offers a framework to facilitate effective communication about family building between patients with inherited cancer risk and their partners.

**Innovation:**

This intervention is the first conversational tool designed for at-risk patients and their partners.

## Highlights

• At-risk patients of reproductive age and their partners have unique concerns.
• This intervention is a tool developed for at-risk patients and their partners.
• The tool encouraged communicated perspective taking behaviors.
• The tool helped couples identify (dis)concordant concerns and next steps.
• The pilot intervention was feasible and acceptable.

## 1 Introduction

According to the American Society of Clinical Oncology (ASCO), the identification and management of individuals with inherited cancer risk (ICR) is a core component of oncology care [1]. Genetic testing for cancer predisposition is utilized to identify individuals at significant increased risk to develop hereditary cancer, enabling individuals to engage in prevention and treatment strategies to reduce cancer incidence and mortality [[2], [3], [4]]. There are several hereditary cancer predisposition syndromes including Hereditary Breast and Ovarian Cancer (HBOC), Lynch, Familial Adenomatous Polyposis, Cowdens, Multiple Endocrine Neoplasia, and Li-Fraumeni. The prevalence varies depending on the pathogenic variant yet the most common are HBOC Syndrome (1 in 400) and Lynch Syndrome (1 in 279) [5,6]. For HBOC Syndrome, for example, the lifetime breast cancer risk of a *BRCA1* carrier is 65% and 60% for *BRCA2* carrier [7]; for Lynch Syndrome, the lifetime colorectal cancer risk for *MSH2* and *EPCAM* carriers is between 33-52% [8].

The increase of genetic counseling and testing to inform the prevention and treatment of hereditary cancer has led to situations where a person is identified as having a pathogenic variant—referred to henceforth as at-risk patients. At-risk patients of reproductive age and their romantic partners have unique challenges and concerns [9,10]. For one, the at-risk individual must navigate cancer risk management (e.g., prophylactic surgery), and associated implications for family building [9,11,12]. In this study, family building involves the act and process of making decisions about children, which could include timing, method/approach, and number [9]. Additionally, these couples may also be concerned about the ICR for future offspring as carriers have up to a 50% risk to pass on their pathogenic variant to each future offspring [9,13,14]. As such, effective communication between couples is critical for cancer risk management and decision making about family building (e.g., timing, method or approach to conception, number of children, adoption, 3^rd^ party family building). Yet, at-risk patients and their partners often experience significant communication challenges when making family building decisions [[15], [16], [17]]. For example, when communicating their desires and preferences, beliefs about passing on the pathogenic variant to possible offspring creates tension and conflict between at-risk patients and their partners [18,19]. Furthermore, discussing family building options takes time and can result in emotional decision-making [9,20].

Given these unique challenges, at-risk patients and their partners require extra time and effort to engage in communicated perspective taking [21]. *Communicated perspective taking* (CPT) is the process by which a person effectively conveys verbally (e.g., confirming other’s identity or perspectives) and nonverbally (e.g., nodding, creating space for others to share their thoughts, ideas, and feelings) that they have understood the other person’s perspective [[21], [22], [23]]. This is in contrast to perspective taking, which is the psychological process of putting oneself into another person’s shoes. Thus, CPT specifically focuses on perspective taking as an interactional process [22,24].

To date, most interventions guided by CPT focus on longitudinal storytelling methods (i.e., how CPT functions during the act of telling stories over time) [21,22,25,26]. Interventions predominantly focus on storytelling among immediate family relationships (e.g., couples, parent(s)-child(ren)) [22,[26], [27], [28]], except for two studies. One focused storytelling among friend dyads [21], and the other was a pilot intervention for an online support group for bereaved parents [29]. With respect to health outcomes, interventions primarily explored the effect of CPT on interactants mental health. Specifically, higher levels of CPT have been associated with improved psychosocial well-being [22,25].

At-risk patients and their partners often struggle navigating the emotional (e.g., wanting to become parents) and clinical aspects (e.g., prophylactic surgeries/interventions) of family building and ICR [9]. CPT, therefore, offers a framework to help at-risk patients and their partners discuss their concerns about having children and ICR in three ways. First, encouraging couples to engage in higher levels of CPT can reduce or maintain low levels of stress during these difficult conversations. Second, conversations about ICR and family building are ongoing, and couples will engage in CPT over the course of months or years as they build their family. Finally, encouraging positive CPT behaviors can help couples reach a concordant perspective on family building options (i.e., being on the *same page*) in order to identify next-steps for family building decision-making [27,30].

To assist couples in communicating about these challenges and concerns, this pilot intervention posits a framework for facilitating communication about family building between at-risk patients and their partners. Most shared decision-making interventions focus on patients and clinicians (e.g., Question Prompt Lists) [31]. Although research suggests clinicians may discuss reproductive health and fertility with at-risk patients, [30] partners are often left out of these conversations [9,[32], [33], [34]]. However, family building decisions implicate both the at-risk patient and their partner; therefore, shared decision-making is more appropriately conceptualized as an intersubjective relationship between the at-risk patient and their partner with their clinicians [35].

Designed to facilitate communication between at-risk patients and their partners, this pilot intervention utilized a discussion task method to help identify family building concerns and encourage CPT behaviors, in light of ICR. Additionally, a structured discussion task was used as most family building conversations tend to occur haphazardly [17,36].

The purpose of this manuscript is to report the feasibility and acceptability as well as explore the impact of the pilot longitudinal, structured discussion intervention. Specifically, we collected data on recruitment and retention, as well as whether couples felt positively toward the intervention, were satisfied, and whether they found the intervention helpful. These assessments were measured through recordings of the discussion task, post-questionnaire responses, and open-ended, dyadic interview feedback.

## 2 Methods

The University of South Florida’s Institutional Review Board (Pro00042279) and Moffitt Cancer Center’s Scientific Review Board (MCC 20310) reviewed and approved this study. This study was a single-arm pilot involving structured discussions tasks with dyadic interviews completed 2-3 months apart.

### 2.1 Recruitment

Our recruitment goal was 15 couples based on expectation of saturation. A recent systematic review of qualitative research studies reporting saturation found when conducting interviews saturation is usual met between nine and 17 interviews [37]. In this study, saturation was reached after nine couples were recruited and completed study procedures. Participants were initially recruited through a clinic at a large cancer center beginning in January 2020. Eligible at-risk patients were identified by a genetic counselor who then contacted those patients via mail and/or email sharing the study’s information and study coordinator’s contact information. Yet after two months with no enrollment, recruitment efforts were switched to social media (i.e., Twitter, Facebook, Reddit) and snowball sampling in March 2020, the beginning of the COVID-19 pandemic [38]. Specifically, recruitment flyers were posted to seven subreddits for individuals with ICR between March 3^rd^ and March 30^th^. Subreddits are subforums dedicated to different topics (such as ICR) on the social media site, Reddit. Two study team members also shared recruitment flyers via Twitter. Additionally, the first author also sent an email to past participants from other studies who consented to being contacted about future research opportunities in early April. Finally, the first author posted recruitment materials to four Facebook groups in late April. After reaching our recruitment goal, an additional 22 at-risk patients expressed interest in the study. Thus, all participants were recruited over 3 months during the first major surge of the COVID-19 pandemic in the U.S.

Interested participants contacted the study coordinator and second author via email who sent a link to an online survey to confirm eligibility, provide contact information, and schedule interviews. Eligible participants: (1) were 18-39 years-old, (2) had received positive genetic test results for a hereditary cancer predisposition syndrome (e.g., Hereditary Breast and Ovarian Cancer (HBOC), Lynch, Familial Adenomatous Polyposis, Cowdens, Multiple Endocrine Neoplasia, Li-Fraumeni); (3) were cohabiting with a partner (i.e., spouse or romantic partner), and (4) had not completed family building.

### 2.2 Intervention development

The intervention was developed by drawing on relevant literature about ICR and family building, using CPT approach, and following the structured discussion task method [17,21,22,26,36,39]. Additionally, the study team’s expertise was integral to development. The study team included a health communication scientist with expertise in the experiences of patients with inherited cancer risk, a behavioral medicine scientist with formal training in health behavior intervention development, delivery, and evaluation, an educational psychologist with formal training in health psychology and biomedical ethics, a social health psychologist with experience in utilizing the structured discussion task method, a certified genetic counselor, and two graduate students trained in health communication.

### 2.3 Data collection

At Time 1 (T1) and Time 2 (T2), couples gave informed consent. The intervention was conducted by a trained study team member consisting of a structured discussion task and follow-up dyadic interview. Data collection began in late April 2020 and finished in August 2020. All couples completed study procedures for T1 and T2 via distance communications (e.g., video conferencing or by phone).

#### 2.3.1 Intervention procedure: time 1

To begin, participants were provided context for the discussion task (e.g., many couples in their situation struggle with family building decision-making). T1’s discussion task was intended to initiate structured conversations about family building decision-making considering ICR. First, participants individually listed their top three concerns regarding family building and ICR on a sheet of paper and rank-ordered them from 1 (most important) to 3 (less important). They were then directed to share their concerns with one another for roughly ten minutes. Questions were provided to guide couples’ conversations such as “What are your thoughts/feelings surrounding the concerns?” and “What might you do to address your concerns together?” The study team member then muted their audio and left the room where study procedures were completed to allow couples to complete the discussion task privately. After roughly ten minutes, the study team member re-entered the room and unmuted their audio. Participants completed a two-minute, online post-discussion questionnaire. Couples then completed a dyadic interview together to gather feedback on the discussion task experience and provided suggestions for improving the discussion task in the future. Participants were asked how the discussion task went, lingering questions about family building and ICR, if they had enough information for future family building decisions, and suggestions for a resource/intervention to help couples make family building decisions given their ICR. To show appreciation and respect for time, participants were compensated $60 for T1’s discussion task and the dyadic interview.

#### 2.3.2 Intervention procedure: time 2

Two to three months later, participants completed procedures for T2. T2 prompted couples to identify and discuss topics about family building decision-making that were (a) easy, (b) difficult, and (c) a topic they disagreed on or caused conflict. The study team member muted their audio and left the room. After roughly ten minutes, the study team member re-entered the room and unmuted their audio. Participants completed the same two-minute, online post-discussion questionnaire from T1. Couples then completed a dyadic interview, where they were asked if the discussion task was helpful and to provide suggestions for future resources and interventions/tools. Again, participants were compensated $60 for T2’s discussion task and dyadic interview.

Discussion tasks and dyadic interviews were audio recorded, transcribed verbatim, and reviewed for accuracy. T1 discussion tasks lasted between 9 and 15 minutes (*M* = 11, *SD* = 1.58). T1 dyadic interviews lasted between 15 and 56 minutes (*M* = 35 minutes, *SD* = 11.62). T2 discussion tasks lasted between 9 and 12 minutes (*M*= 11, *SD* = .85). T2 dyadic interviews lasted between 16 to 72 minutes (*M* = 34, *SD* = 16.65). Participants were assigned pseudonyms.

### 2.4 Measures

Prior to discussions, participants were asked to complete basic demographic questionnaires, assessing their age, sex/gender, race/ethnicity, education, employment/income, religious affiliation, insurance status, relationship length and family. After each structured discussion task, participants were independently asked specific questions regarding their perceptions of the interaction with their partner (10 items) and their emotional response (nine items). Interaction items were drawn from previous research [40,41] and included perceptions of disclosure, intimacy, influence, understanding, validation, acceptance, and emotional valence of the interaction. Emotional items were adapted from the Positive and Negative Affect Scale [42] to reflect emotions across two emotional dimensions of valence and activation. Each item was rated on a Likert-type scale ranging from 1 (very little) to 5 (a great deal).

### 2.5 Data analysis

Recruitment and retention data as well as demographic data and interaction items were summarized using descriptive statistics. Additionally, under the guidance of the first author trained in qualitative methods, the second author thematically analyzed the discussion task and dyadic interview data in two ways [43,44]. First, couples’ discussion topics at T1 (concerns) and T2 (easy, difficult, and conflict/disagreement provoking) were analyzed through a two-step process. The second author began by revisiting each verbatim transcript to note each participant’s topics. Next, the corpus of topics were reviewed (e.g., having a child before graduation, having a child in the next year) to identify overlapping ideas (e.g., family building timeline) [45]. Throughout this process, the second author met with the first author to discuss what emerged from the data and refine the coding following procedures for data conferencing [46,47]. Data conferencing is a tool for qualitative data analysis verification where the analyst writes up brief reports on coding procedures including exemplar quotations, which are shared with a scholar who was not part of the analysis process to assess the reliability of coding procedures (i.e., the degree to which exemplars fit within the described coding procedures) and analytic findings. As a means for further verification, throughout the data collection and analysis, the authors kept track of recruitment, methodological decisions, coding, and analyses in a reflexivity journal [48].

Second, to evaluate the utility of the discussion task interventions, the second author analyzed (a) post discussion task questionnaire results from T1 and T2 and transcripts from (b) the discussion tasks and (c) dyadic interviews. Specifically, transcripts were uploaded, organized, and coded in MAXQDA2020. An applied thematic analysis [49] was conducted following a “selective approach” [50,51] to qualitative data analysis on discussion task and dyadic interview data from T1 and T2. The second author became familiar with the data by listening to all discussion task *and* dyadic interview recordings and reading and re-reading each transcript several times. Next, data were split roughly in half for T1 (*n* = 8) and T2 (*n* = 7) and initially coded (i.e., noted “interesting feature[s] of the data,”;[44], p. 87; e.g., “learning about partners FB flexibility”). Memos were kept to record coding decisions and track potential theoretical insights [49]. After coding the first half of the data, initial themes were generated by organizing and collapsing codes (e.g., *same concerns/different order*; same concerns/same order”) into one another around identified concepts (e.g., *concordant concerns)* [49]. These initial themes were then checked across remaining data from T1 (*n* = 7) and T2 (*n* = 6) to refine and bring each theme into conversation with one another.

## 3 Results

### 3.1 Feasibility

Feasibility was assessed in two ways: (1) recruitment success and (2) intervention completion. Although initial recruitment through clinic referral was not successful, the target sample was recruited within two months after moving to online and snowball sampling recruitment methods. Fifteen couples participated in this longitudinal study at T1 (May-June 2020) T1 (*n* = 30), and 13 couples participated at T2 (July-September 2020). Two couples withdrew from the study at T2 because of a change in fertility status which made them ineligible to complete the project. For detailed demographics of participants see Table 1.

**Table 1:** Demographics.

Individual level variables		Total (*n* = 30)		
		*Min-Max*	*M*	*SD*
Age		22-37	31.03	3.03
		Freq	%	
Sex	Female	15	50.00	
	Male	15	50.00	
Gender	Female	15	50.00	
	Male	15	50.00	
Race/Ethnicity⁎	American Indian/Alaska Native	2	6.67	
	Asian	1	3.33	
	Black/African American	2	6.67	
	White/Caucasian	27	90.00	
	Jewish	1	3.33	
Hispanic/Latinx	Yes	2	6.67	
Highest Education	Some college or vocational school	3	10.00	
	College graduate (4 years)	9	30.00	
	Some graduate or professional school	1	3.33	
	Graduate or professional degree	17	56.67	
Employment	Full-Time	24	83.33	
	Part-Time	2	6.67	
	Not Employed	4	13.33	
Religious Affiliation	Jewish	6	20.00	
	Catholic	3	10.00	
	Protestant	3	10.00	
	Non-denominational Christian	1	3.33	
	No religious affiliation	17	56.67	
Financial Situation	Not very good	2	6.67	
	Comfortable	16	53.33	
	More than enough	11	36.67	
Health Insurance (last 12 months)	Yours or someone else’s job or union	29	96.67	
	Medicaid	1	3.33	
	Traditional fee-for-service	1	3.33	
Type of Insurance Plan	Preferred Provider organization	19	63.33	
	Health maintenance organization	3	10.00	
	Medicaid	1	3.33	
	Tricare (Military)	2	6.67	
	EPO	1	3.33	
	Don’t know	3	10.00	
Uninsured (last 12 months)	Yes	4	13.33	
	No	26	86.67	

Couple Level variables				
Relationship Status	Dating	2	13.33	
	Married	13	86.67	
Annual household income	$50,000-$74,999	3	20.00	
	$75,000 or more	12	80.00	
Number of children	1	3	20.00	
	No children currently	12	80.00	
		*Min-Max*	*M*	*SD*
Length of the relationship		1.75-14	7.8	3.59

^⁎^ Participants were asked to select all that applied

For the T1 discussion task, participants rank-ordered their concerns regarding family building decision-making and ICR. At T1, one participant listed two instead of three concerns. For the T2 discussion task, couples listed a topic that: (a) was easy to discuss, (b) was difficult to discuss, and (c) caused conflict/disagreement. At T2, a few couples noted in interviews having a difficult time coming up with concerns that caused conflict/disagreement.

### 3.2 Acceptability

Acceptability was assessed in two ways: (1) through dyadic interviews to determine if participants liked the intervention and found it helpful and (2) post-questionnaires to determine participants’ perceptions of their interaction with their partner as well as their emotional responses. Overall, participants noted the usefulness of the pilot intervention. Although most participants said they had previously talked about their concerns, they appreciated the organized nature of the structured discussion task. For example, Ciara (32, at-risk patient) said,

*I think the structured setting, was good to just keep us on, you know, focused on it.*

Similarly, Emmet (32, at-risk patient) stated,

*Having the time for structured conversations is really cool. It’s different. Usually these things come up haphazardly, so when you really put the time into something to talk about it, you end up more focused, which is nice.*

Nilesh (26, partner) further explained how the process of the structured discussion task was helpful in encouraging thorough discussions. He shared,

*It [the structured discussion task] was a nice, because it makes you write things down, and then actually have to think through it while you’re talking to each other. So, it gives like hard points to discuss. So, it was pretty nice versus just talking in general, and then somethings that we should discuss, probably not getting brought up because they weren’t written down, or you didn’t have to think about the question before talking about it instead of just like having a regular conversation. So, it was helpful.*

Moreover, participants reported that the structured discussion task facilitated a more effective conversation. For instance, Allyson (28, at-risk patient) explained that in addition to the dedicated time, the structure of the discussion task enabled her partner to better engage in effective CPT behaviors by giving him a dedicated time to actively listen to her concerns. She said,

*I also think it was nice because I feel like I often initiate these conversations. So, it's kind of nice to have dedicated time where we both had to be totally active in the conversation versus needing to bring stuff up and hoping he’s listening.*

As Cole (37, partner) concluded,

*The conversation in general was actually pretty productive.*

In short, couples liked the intervention and found the process of the intervention useful when discussing family building options in light of their ICR.

Additionally, post-questionnaire results at both time points revealed the intervention was not stressful, and participants felt relatively high levels of intimacy and disclosure. For the post discussion questionnaire at T1, participants reported that the discussion was overall low stress (*M* = 2.02) (See Table 2), and they felt relatively high levels of intimacy (*M* = 4.27) and disclosure (self *M* = 4.27; partner *M* = 4.23) (See Table 3). For the post discussion questionnaire at T2, participants also reported that the discussion was overall low stress (*M* = 1.9) (See Table 2), and they felt relatively high levels of intimacy (*M* = 4.27) and disclosure (self *M* = 4.31; partner *M* = 4.31) (See Table 3).

**Table 2:** Feelings following structured discussion task at T1 and T2⁎

Assessment question	T1 (*n* = 30)		T2 (*n* = 26)	
	M	SD	M	SD
Sad	1.83	.87	1.65	.78
Excited	2.37	.85	2.23	.95
Interested	3.00	.70	3.12	.82
Frustrated	1.53	.78	1.54	.76
Stressed	2.20	.96	1.92	.85
Anxious	2.37	.72	1.92	.85
Upset	1.40	.56	1.35	.63
Alert	2.57	.73	2.38	.85
In control	2.23	.82	2.60	.76

^⁎^ Responses scaled from 1 (not at all) to 4 (very much)

**Table 3:** Assessment of structured discussion task at T1 and T2.⁎

Assessment question	T1 (n = 30)		T2 (*n = 26)*	
	*M*	*SD*	*M*	*SD*
Intimacy	4.27	1.05	4.27	.78
I disclosed	4.27	.64	4.31	.97
My partner disclosed	4.23	.77	4.31	1.01
Influenced the conversation⁎⁎ (1= more me, 5 = more them)	2.93	.84	3.04	.66
Made me feel understood	4.50	.94	4.23	.95
Made me feel validated	4.47	.82	4.12	1.07
Made me feel accepted	4.60	.86	4.54	.76
Resulted in positive feelings	4.30	.70	4.00	1.10
Resulted in negative feelings	1.77	.77	1.96	1.00
Resulted in mixed feelings	2.03	.89	2.19	1.27

^⁎^ Responses scaled from 1 (very little) to 5 (a great deal)

^⁎⁎^ One participant chose not to answer this question (n = 29) for this question.

### 3.3 Outcomes

In terms of the pilot intervention’s outcomes, analysis revealed three themes. First, at-risk patients and their partners noted that the discussion task helped them realize *concordant concerns* about family building and ICR. For many couples, prior to participating in the study, conversations about family building and ICR began organically (i.e., during casual daily conversations). In contrast to these organic conversations, participants in this study stated that the discussion task helped them discuss each other’s perspectives on family building options and *how* and *why* certain options were prioritized and vital to future family building decisions (e.g., adoption, IVF with/without PGD, no intervention). For example, Jackson (30, partner) said,

*It was kind of ironic that we had the same concerns, but maybe it’s because we talk about it so frequently.*

Second, participants noted that the discussion task prompted them to discover and/or confront *discordant concerns* about family building and ICR. Discovering discordant concerns sometimes resulted in highlighting relational conflict, suggesting low levels of CPT. For instance, Christine (34, partner) explained that it was during the discussion task she learned about “All of his concerns.” Reflecting on the discussion task’s utility, she said:

*It’s 10 minutes to tell your side of the story, and how are you supposed to just respond to those? I feel like a lot of it is just hearing the other one’s concerns, which is great because it’s the first time he’s ever actually told me those things, because he doesn’t particularly talk to me about his feelings a lot, if hardly ever, which is helpful, but I’m not sure it’s going to solve anything.*

Here, Christine notes that the current time constraints did not provide her with enough time to share her perspective, hear her husband’s newly disclosed perspective, and respond (i.e., CPT) effectively. Thus, discovering discordant concerns in the context of a 10-minute, structured discussion task might produce the unintentional outcome of highlighting distal relational tensions (e.g., not disclosing one’s feelings to their partner) without the intervention providing additional resources or time to work through that conflict.

However, discovering discordant concerns was not always negative. For some participants, learning about their discordant concerns allowed them to take note of their partners unique perspectives and identify new things to consider. For instance, Nathan (30, partner) shared with the interviewer:

*Jennifer’s last point was one that I hadn’t thought about. Not really something that I learned about Jennifer, but another…concern that she has that we haven’t talked about. And that was her getting sick before we were able to finish having the children that we wanted – specifically the number of children.*

Thus, because his wife shared her personal concern about developing cancer before completing family building, Nathan learned and discussed new things regarding her perspective.

Finally, participants noted that the task helped them *work through* and *agree upon* next steps for having children. Lindsey (30, at-risk patient) reported that during the discussion task she and her husband:

*Identified a couple next steps to take in terms of what we want to do and to learn more about family planning about reducing genetic risk—[the] risks and benefits. We talked about wanting to talk to my doctor, and doctor and gathering more information.*

Her husband, Matthew (31, partner), followed up saying,

*[The discussion task] gave us an opportunity to really be on the same page about a topic that doesn’t come up. Like what should we do about the potential genetic risk to our potential future child?*

Thus, the structured discussion task about concerns centered around family building and ICR, allowing couples to take stock of (dis)similar concerns and jointly identify next steps for family building.

## 4 Discussion

This study describes the feasibility, acceptability, and outcomes of a pilot intervention designed to facilitate communication between at-risk patients and their partners regarding family building decision-making. Results indicate this intervention is feasible and acceptable, providing a framework for facilitating communication about family building decisions in light of ICR.

### 4.1 Recruitment and Retention

Following two months of unsuccessful clinic recruitment, recruitment efforts rerouted to social media (i.e., Twitter, Facebook, Reddit) and snowball sampling [38]. Social media recruitment is often more successful at enrolling adolescent and young adult cancer survivors for fertility related research than traditional clinic-based recruitment, [52] and this study retained all couples across two points in time except two who had to withdraw at T2 due to changes in fertility status. As noted earlier, an additional 22 at-risk patients expressed interest in the study during active recruitment; however, we had already reached saturation at nine couples and still enrolled six more couples to confirm saturation. Online forums and support groups are also important places for patients and partners to find support and seek information about ICR [[53], [54], [55]]. Nevertheless, important group differences occur between social media and clinic recruited samples (e.g., race, treatment, mental health) [52]. These findings illustrate the utility of social media recruitment strategies [56], but demographic concerns must be considered. Additional efforts during recruitment (e.g., utilizing recruitment flyers depicting racially diverse and same-sex couples) might improve the representativeness of these samples, yet the overall lack of representation among diverse population likely goes beyond recruitment efforts, demonstrating health disparities for cancer genetic services [57,58].

### 4.2 Completing the discussion tasks

For T1, at-risk patients and their partners were easily able to rank-order their top three concerns regarding family building decision-making and ICR. Participants struggled more with T2’s prompt—finding it difficult to identify areas of disagreement. This could be due to overarching agreement about wanting to have a child or children being more salient than possible disagreements [59].

### 4.3 Feedback on and impact of the intervention

In addition to this study revealing that the discussion task did not create stress, participants found the intervention useful and were satisfied. Specifically, they liked that the structured discussion task provided time to discuss their family building concerns as well as discuss how their family building preferences were concordant or discordant with each other. For some, this intervention presented an opportunity to hear and consider each other’s concerns. For other couples, the discussion task guided at-risk patients and their partners to work through making decisions about family building. Couples who had concordant perspectives on family building options were able to be thinking through problem solving likely because they were on the same page [30]; whereas couples who had discordant perspectives needed more time to think through and process their partners’ concerns and work toward getting on the same page about family building options [[15], [16], [17]]. Thus, this pilot intervention provides at-risk patients and their partners not only with time but also guidance for having these difficult conversations.

### 4.4 Innovation

This conversational tool is the first developed for at-risk patients and their partners, answering a recent call for researchers in HBOC to “investigate the utility of clinical tools such as conversational guides” (p. 495) [39]. This pilot intervention is innovative in three ways. First, it includes partners of at-risk patients as instrumental stakeholders in reproductive health decision making. Second, it provides structure and dedicated time to assist couples in having these difficult conversations. Third, it offers novel contributions to CPT theorizing. Specifically, the study’s findings that the discussion task helped at-risk patients and their partners work through and agree upon next steps for having children suggests CPT may not only have an impact on individual’s psychosocial wellbeing, but it may also influence actions individuals take when communicating in the future about family building. That is, by giving participants a task forcing them to interact with some structure (i.e., dedicated time and sequence of the conversation) and resources that might facilitate CPT (i.e., listing concerns so they are not forgotten, providing discussion questions that encourage individuals to share their perspective and respond to their partners’ perspective in kind), it may be possible to assess how CPT influences well-being (i.e., stress) as well as other health behaviors (i.e., identifying next steps). In short, this tool facilitates communication between at-risk patients and their partners, which may ultimately provide structured support for healthcare decision making.

### 4.5 Limitations

There are limitations to this work. First, this sample is homogeneous in terms of race/ethnicity (90% white), socioeconomic status, insurance status, heterosexual couples, and HBOC Syndrome, which limits its generalizability. Relatedly, the at-risk patient was a male and the partner a female in only one couple, which may also limit acceptability and outcome conclusions. Further refinement and testing are needed to assess acceptability with more diverse populations. Second, participants recruited from online ICR-related communities (e.g., support forums) and clinical populations (e.g., those seeking a genetic counselor) might be more motivated to have these discussions than couples with ICR who do not use these online resources. Finally, as couples volunteered for this study, participants may be more aware of the need to communicate effectively about family building and ICR; thus, future testing of this intervention should consider the possible impact of couples’ communication skills.

## 5 Conclusions

This study provides a pilot intervention to facilitate communication about family building between at-risk patients and their partners. To our knowledge, this is the first intervention designed to guide these couples’ communication and decision-making about family building considering ICR. Future studies with larger sample sizes will assess efficacy of this intervention in assisting couples’ family building decision-making and improving their communication satisfaction as well as their CPT ability [21,24]. Furthermore, future research with other key stakeholders (e.g., genetic counselors, oncologists, reproductive endocrinologists, nurse practitioners/physician assistants) who deliver care to this population and partnerships with patient support/advocacy organizations will be important to help identify the best modality, timing, and approach for this intervention.

## Funding

Marleah Dean, PhD was supported by an Institutional Research Grant, IRG-17-173-22 from the 10.13039/100000048American Cancer Society.

## Author contributions statement

*Marleah Dean*: Conceptualization, Data curation, Formal analysis, Funding acquisition, Investigation, Methodology, Project administration, Supervision, Writing - original draft, Writing - review and editing.

*Jonathan T. Baker*: Data curation, Formal analysis, Investigation, Methodology, Project administration, Writing - original draft, Writing - review and editing.

*Maija Reblin*: Conceptualization, Funding acquisition, Investigation, Methodology, Writing - original draft, Writing - review and editing.

*Elizabeth A. Hintz*: Conceptualization, Data curation, Funding acquisition, Investigation, Methodology, Writing - review and editing.

*Carolyn Haskins*: Investigation, Methodology, Writing - review and editing.

*Susan T. Vadaparampil*: Conceptualization, Funding acquisition, Investigation, Methodology, Supervision, Writing - review and editing.

*Gwendolyn P. Quinn*: Conceptualization, Funding acquisition, Investigation, Methodology, Supervision, Writing - review and editing.

## Declaration of Competing Interest

The authors report no conflicts of interest.
